# Tibiopedal and distal femoral retrograde vascular access for challenging chronic total occlusions: predictors for technical success, and complication rates in a large single-center cohort

**DOI:** 10.1007/s00330-020-07082-3

**Published:** 2020-07-28

**Authors:** Gerd Grözinger, Jan Hallecker, Ulrich Grosse, Roland Syha, Dominik Ketelsen, Klaus Brechtel, Mario Lescan, Konstantin Nikolaou, Christoph Artzner

**Affiliations:** 1 Department of Diagnostic and Interventional Radiology, University Hospital of Tübingen, Hoppe-Seyler-Strasse 3, 72076 Tübingen, Germany; 2 Ihre-Radiologen.de MVZ GmbH, Interventional and Diagnostic Imaging Centers, Heinz-Galinski-Strasse, 13347 Berlin, Germany; 3 Department of Diagnostic and Interventional Radiology, Prosper Hospital Recklinghausen, Mühlenstrasse 27, 45659 Recklinghausen, Germany; 4 Department of Thoracic and Cardiovascular Surgery, University Medical Center Tübingen, Tübingen, Germany

**Keywords:** Angioplasty, Arterial occlusive diseases, Arteriosclerosis, Peripheral arterial diseases

## Abstract

**Objective:**

To evaluate the safety and effectiveness of tibiopedal and distal femoral access for retrograde crossing of chronic total occlusion (CTO) in Rutherford stage III to VI peripheral arterial occlusive disease, and to determine factors that correlate with technical success.

**Material and methods:**

One hundred seventy-one consecutive patients were included in this retrospective study. Rutherford stages were III, IV, and V/VI in 24%, 8%, and 67% of patients. Inclusion criteria were CTO at the superficial femoral (SFA), popliteal (PA), and/or below-the-knee (BTK) level, and a failed antegrade treatment followed by a distal retrograde approach. The numbers of occluded vascular levels (OVL), lesion length, degree of calcification, technical success rate, complications, and clinical outcome were noted.

**Results:**

OVL were 1 in 72%, 2 in 20%, and 3 in 8% of patients. CTOs were longer than 20 cm in 45.6% of cases and showed severe calcifications in 50.3%. Target vessels for distal access were the distal SFA/PA in 17% and BTK in 83%. The overall technical success rate was 82%. Severe calcification decreased technical success (*p* = 0.01) despite lesion length and Rutherford stage. Clinical outcome improved in 123/152 patients with a significant increase of the median ABI (*N* = 158) from 0.53 (interquartile range 0.39 to 0.61) to 0.85 (0.59 to 1.03; *p* < 0.001). Complications were reported in 7.6% cases with 2.3% related to the distal vascular access.

**Conclusion:**

The tibiopedal and distal femoral retrograde access presents a safe and effective treatment option of CTOs at the thigh and/or BTK after a failed antegrade attempt improving clinical outcome. Technical success decreased with lesion’s degree of calcification.

**Key Points:**

*• Safety and effectiveness of the tibiopedal and distal femoral access for retrograde crossing of chronic total occlusion.*

*• Target lesion’s degree of calcification decreases technical success.*

*• Complications related to the distal vascular access were rare.*

## Introduction

Peripheral arterial occlusive disease (PAOD) poses an expanding healthcare challenge due to a global increase in the aging population and a growing number of patients with diabetes [1–3]. An unmet need exists for interventional vascular treatment, especially in Rutherford stage IV to VI patients where amputation rates are still high [4]. In recent years, endovascular treatment strategies have moved beyond the standard aortoiliac and femoropopliteal revascularization [5, 6] and treatment of below-the-knee (BTK [including segment P3 of the popliteal artery]) vessels in patients with critical limb ischemia (CLI) [2, 5] to include more aggressive handling of patients with femoropopliteal chronic total occlusions (CTO). Among a growing number of dedicated devices for CTO revascularization [5, 6], a retrograde approach with tibiopedal access is an increasingly used strategy [5, 7]. Since its first usage, the techniques and dedicated material have improved, and technical success rates from 80% up to 100% for lesion crossing have been reported [8–14]. The application of this technique has been expanded to patients with lifestyle-altering claudication (Rutherford stage III) accompanied by an increase in the technical success rates of treatment [8, 9, 15]. Yet, some concerns remain regarding the success rates and safety of this technique in patients with Rutherford category III [7].

This retrospective study aims (A) to evaluate the hypothesis that the use of the tibiopedal and distal femoral approach for retrograde crossing of CTOs is an efficacious and safe procedure independent of PAOD stage (Rutherford III to VI) in cases where an antegrade approach has failed, and (B) to determine anatomical and technical factors for the prediction of technical success.

## Material and methods

### Patients

One hundred and seventy-one consecutive patients were included in this HIPAA-compliant, IRB-approved, retrospective study between January 2011 and September 2019 with waiver of informed consent. The patient cohort comprised 51 women (29.4%) and 120 men (70.6%) with a mean age of 74.1 ± 9.7 years. Consecutive patients refer to patients with a failed antegrade approach and a target vessel suitable for distal puncture at the level of the distal thigh or lower leg, which accounts for 2.36% (171 of 7254) of all patients treated for peripheral arterial disease at the center. Additional baseline characteristics including vascular risk factors and PAOD stage are listed in Table 1a.

**Table 1:** Baseline characteristics of (a) patients and (b) lesions

(a) Baseline characteristics of patients	
	*N* (%)
Number of patients	171
Age	74.1 ± 9.7
Gender male	120 (70.6%)
Vascular risk factors	
Hypertension	130 (76.0%)
Diabetes mellitus	93 (54.4%)
Smoker (current/ex-smoker)	68 (39.8%)
Hyperlipidemia	80 (46.8%)
Coronary artery disease	93 (54.4%)
Chronic renal insufficiency	75 (43.9%)
Obesity	36 (21.1%)
PAOD stage (Rutherford)	
3	41 (24.0%)
4	14 (8.2%)
5/6	116 (67.8%)
(b) Baseline lesion characteristics	
Occluded target vessel (more than one mention)	
SFA	68
POP	56
TTF	41
ATA	46
PA	32
PTA	17
Bypass	6
Multilevel disease occluded segments (SFA/POP/BTK) or combination of segments	
1	123 (71.9%)
2	34 (19.9%)
3	14 (8.2%)
Level of occlusion	
Thigh only	58 (33.9%)
BTK only	75 (43.9%)
Both levels	37 (21.6%)
Degree of calcification ^a^ (0 = none; 1 = mild; 2 = moderate; 3 = severe) *n* = 167*	
0	2 (1.2%)
1	15 (9.0%)
2	66 (39.5%)
3	84 (50.3%)
Total lesion length of the chronic total occlusion	
< 5 cm	22 (12.9%)
5–10 cm	25 (14.6%)
10–15 cm	23 (13.5%)
15–20 cm	23 (13.5%)
> 20 cm	78 (45.6%)

*SFA* superficial femoral artery, *POP* popliteal artery, *TTF* tibioperoneal trunk, *ATA* anterior tibial artery, *PA* peroneal artery, *PTA* posterior tibial artery, *BTK* below-the-knee including segment 3 of the popliteal artery

^a^Calcification severity as determined by angiographic calcium score (*ACS*) and Peripheral Artery Calcification Scoring System (*PACSS*)

*Excluding *n* = 4 by-pass grafts

Forty-one patients (24%) suffered from severe claudication and 130 (76.0%) from critical limb ischemia (CLI). All included patients were referred for endovascular recanalization based on a consensus decision of the institution’s vascular board. Claudicants were included in the study when there was an indication for interventional treatment according to the latest guidelines [16, 17]. All claudicants received a conservative treatment (optimization of medical therapy and structured walking training) before interventional treatment was considered. Interventional treatment was indicated by a multi-specialty clinical conference in consensus. All patients provided written informed consent for the procedure.

Inclusion criteria were (A) a chronic total occlusion (CTO) at the femoral, popliteal, or BTK level, and (B) a failed antegrade approach, e.g., failed crossing or missed distal re-entry into the true lumen after subintimal crossing.

### Endovascular technique

All interventions were performed via femoral access with either an antegrade or retrograde cross-over approach. In the case of retrograde access, a 6-French (F) or 7-F 45-cm cross-over sheath was used (Fortress, Terumo). All patients received 5000 units of intra-arterial heparin at the start of the procedure augmented with additional heparin depending on the duration of the intervention, up to 10,000 units in heavy patients with procedure times exceeding 3 h and risk factors for occlusion of the distal vessel. Vasodilators were applied as needed.

**Fig. 1:**
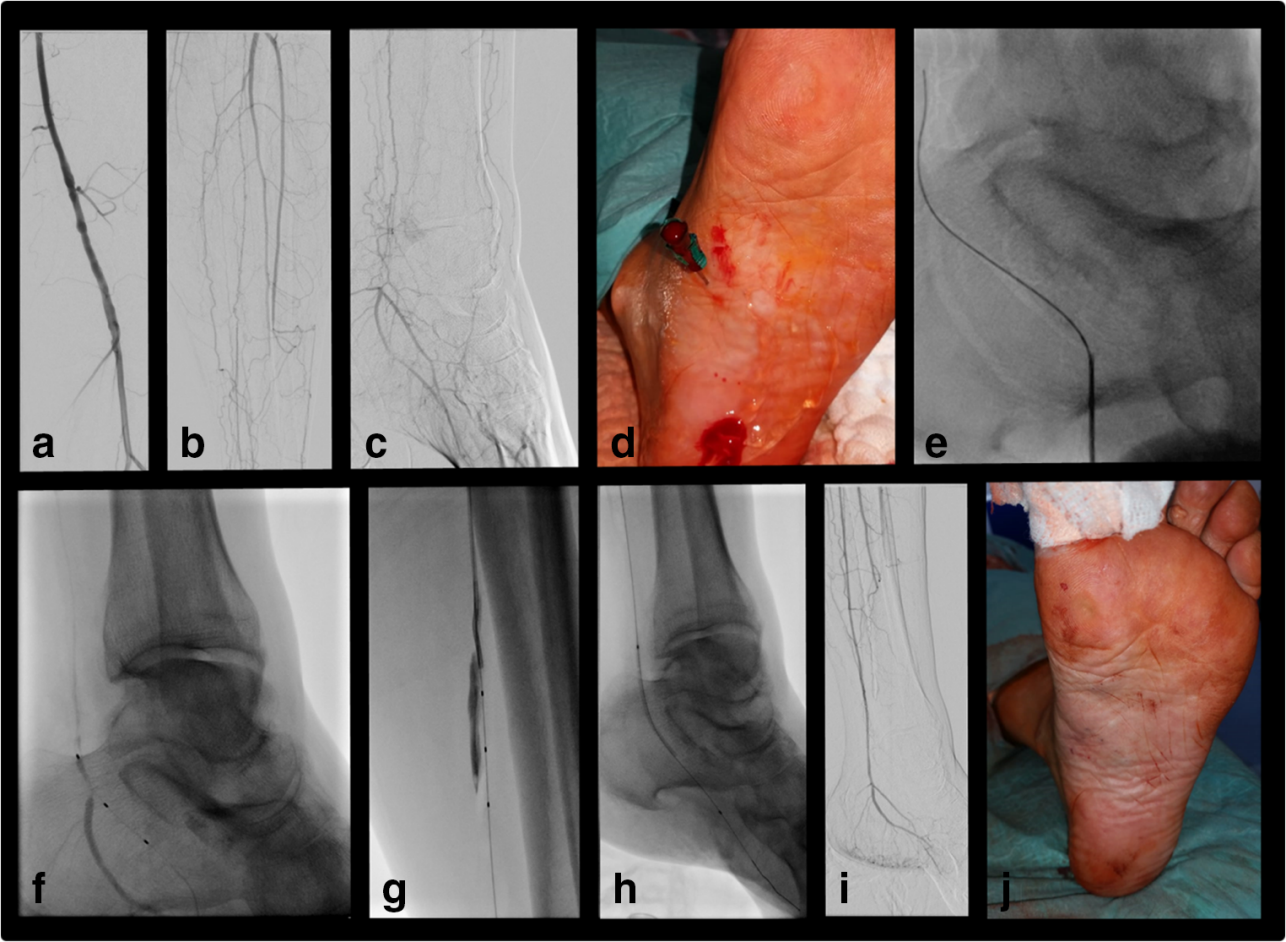
Example of a 74-year-old female patient with peripheral arterial occlusive disease (PAOD) Rutherford stage 5 with ischemic ulcer at Digitus I. **a**–**c** Occlusion of all BTK (below-the-knee) vessels without direct flow path to the foot. Present pedal arteries. Failure of antegrade CTO (chronic total occlusion) passage with antegrade dissection (seen in **g**). **d**, **e** Fluoroscopically and sonographically guided puncture of the proximal medial plantar artery using a 21-G micropuncture needle and a 0.018-in. wire. **f** Advancement of a 2.6-French support catheter (Trailblazer, Medtronic) over the 0.018-in. wire. **g** Successful crossing of the posterior tibial artery CTO from retrograde with subsequent cannulation of the angulated 4-French catheters, which was introduced from an antegrade approach. **h** Conversion to antegrade (approach for treatment) followed by low-pressure percutaneous transluminal angioplasty (PTA) of the puncture site and manual compression. **i** Final angiogram after posterior tibial artery PTA. **j** Photograph of the distal puncture after removal of the distal access

The decision for distal puncture was at the performing physician’s discretion after a failed antegrade approach with prior general approval by the multi-disciplinary clinical conference. The target vessel for distal puncture was visualized by DSA or fluoroscopy with contrast administration given via the antegrade sheath. Distal access was achieved by fluoroscopic guidance as reported previously in the literature [7]. Ultrasound was only utilized in few cases for distal puncture. A typical case is illustrated in Fig. 1. Of note, retrograde puncture of the distal SFA or proximal popliteal artery was performed in the supine position with a puncture from the medial side with the leg in outwards rotation for better practicality compared with the prone position. A 21G micro-puncture needle was used in conjunction with a 0.018-in. wire (principally V-18 ControlWire, Boston Scientific; in a few cases Hi-Torque Command 18, Abbott, or Glidewire Advantage, Terumo) followed by a 2.6-F support catheter (Trailblazer, Medtronicor Seeker CR Bard) when vascular access was achieved. In most cases, lesions were crossed using 0.018-in. CTO wires. In a few cases (*n* = 8), four French sheaths were used from the distal access to facilitate larger crossing devices or 0.035-in. wires. After successful retrograde crossing, the retrograde wire was externalized via the antegrade sheath and a 4-F angiography catheter. Thereafter, prolonged low-pressure PTA in combination with external pressure achieved closure of the distal access site followed by treatment of the CTO from an antegrade approach.

### Procedural outcome assessment

Technical success with regard to access was defined as the ability to gain percutaneous entry into a suitable distal artery and to introduce a support catheter over a wire. The procedure was rated successful when (A) the CTO was crossed and the wire was externalized, and (B) the lesion was treated successfully treated.

The angiogram for each patient was reviewed in consensus by two interventional radiologists with 8 and 11 years of experience to evaluate lesion characteristics. Degree of calcification was assessed referring to two previously published criteria: the Peripheral Artery Calcification Scoring System (PACSS) and the angiographic calcium score (ACS) [18–20]. In accordance with these classifications, lesions were assigned to four groups with a grading of 0 (no calcification), 1 (light calcification), 2 (moderate calcification), and 3 (severe calcification). A number of other features were also assessed, including (A) the diameter of the distal target vessel for puncture, (B) the length of the CTO, (C) the number of occluded vascular levels (SFA and/or popliteal artery and/or BTK arteries or any combination of the latter), and (D) the catheter or sheath size used for distal access.

Treatment success after crossing was noted. Clinical outcome was assessed by ankle brachial index (ABI), improvement of pain-free walking distance (Rutherford stage III), resolving ischemia-related pain (Rutherford stage IV), and healing of wounds (Rutherford stage V/VI). Clinical outcome was assessed within 30 days of the index procedure.

Procedural lesion treatment method and success, runoff status, and long-term clinical outcomes, such as limb salvage, were beyond the scope of this study.

Complications were recorded using SIR and CIRSE guidelines using the patient’s electronic medical record in retrospect [21, 22].

### Lesion characteristics

The lesion characteristics are summarized in Table 1b. The CTO was present in the SFA/P1 in 58, BTK arteries in 75, or both SFA/P1 and BTK arteries in 37 cases. The numbers of occluded vascular levels (SFA and/or popliteal artery [P1 and P2] and/or BTK arteries [including P3]) were 1 level in 123 (72%) patients, 2 levels in 34 (20%) patients, and 3 levels in 14 (8%) patients.

Most CTOs were classified as long lesions with 78 of the cases being longer than 20 cm. There was a variable degree of calcification with 50.3% of cases showing severe calcifications (grade 3). Details are provided in Table 1b.

**Fig. 2:**
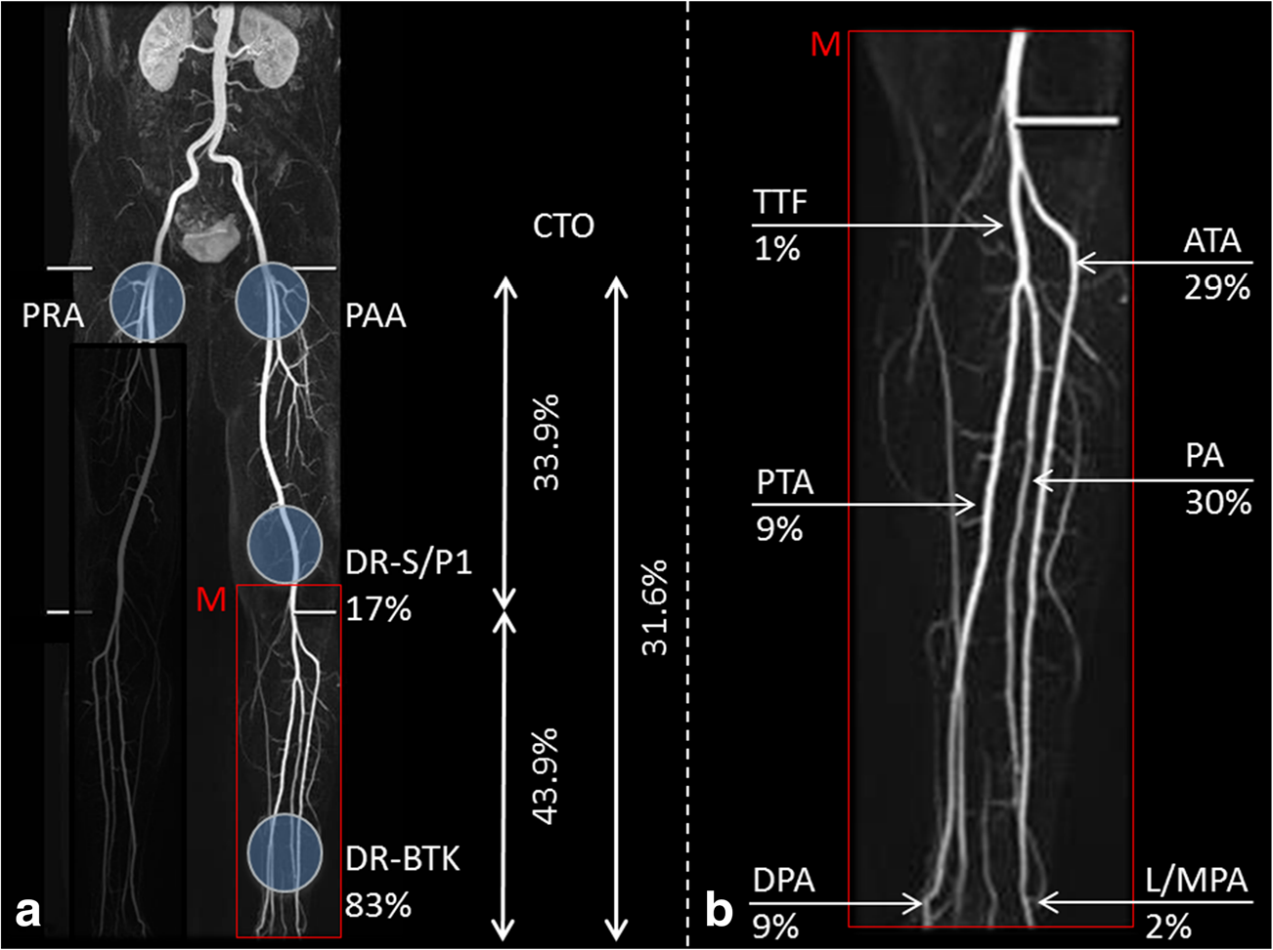
Illustrating pattern of chronic total occlusions (CTO), antegrade and distal retrograde access sites exemplary for left-sided CTO. CTO locations were either limited to the superficial femoral artery (SFA) and proximal popliteal artery (POP), or limited below-the-knee (BTK), or included both. Proximal access site was always the common femoral artery either retrograde cross-over (PRA) for lesions of the proximal SFA or antegrade in all other cases (PAA). Distal retrograde access was gained either by puncture of the distal SFA or proximal POP (DR-S/P1) or BTK (DR-BTK). BTK access sites were the anterior tibial artery (ATA), tibioperoneal trunk (TTF), posterior tibial artery (PTA), peroneal artery (PA), lateral/medial plantar artery (L/MPA), and dorsalis pedis artery (DPA). M is a magnification of BTK

Target vessels for distal access were the distal SFA/P1 and BTK arteries in 29 (17%) and 142 (83%) cases (Table 2). Of the cases where BTK arteries were used as the puncture site, the peroneal artery was used most frequently (52 cases), followed by the anterior tibial artery (50 cases). Details are provided in Table 3 and illustrated in Fig. 2.

**Table 2:** Target vessel for distal access

Target vessel for distal access	Number	Mean vessel diameter
SFA/P1	29 (17.0%)	5.1 ± 0.7
TTF	1 (0.7%)	3.0 ± 0.0
ATA	50 (29.2%)	2.8 ± 0.4
PA	52 (30.4%)	2.7 ± 0.35
PTA	15 (8.8%)	2.5 ± 0.4
L/MPA	3 (1.7%)	1.5 ± 0.0
DPA	21 (12.3%)	2.0 ± 0.3

*SFA* superficial femoral artery, *POP* popliteal artery, *TTF* tibioperoneal, *ATA* anterior tibial artery, *PA* peroneal artery, *PTA* posterior tibial artery, *DPA* dorsalis pedis artery, *L/MPA* lateral/medial plantar artery

**Table 3:** Factors affecting the technical success

Parameter	Technical success *N*/*N*_total_ (%)	*p* value
Number of segments^$^	171	
1	101/123 (82.1%)	0.52
2	29/34 (85.3%)	
3	10/14 (71.4%)	
Lesion length		
< 20 cm	76/93 (81.7%)	0.95
> 20 cm	64/78 (82.1%)	
Degree of calcification*	167	
0	2/2 (100%)	0.01
1	15/15 (100%)	
2	57/66 (86.4%)	
3	62/84 (73.8%)	
PAOD stage (Rutherford)		
2/3	34/41 (82.9%)	0.89
4	11/14 (78.5%)	
5/6	95/116 (81.9%)	
Location for distal access		
Dist SFA/P1	27/29 (93.1%)	0.06
BTK	113/142 (79.6%)	

*PAOD* peripheral arterial occlusive disease, *SFA* superficial femoral artery, *POP* popliteal artery, *BTK* below-the-knee including segment 3 of the popliteal artery

^$^Number of occluded segments (SFA/POP/BTK) or combination of segments

*Degree of calcification referring to PACSS and ACS grading with 0 = none, 1 = slight, 2 = medium, and 3 = severe; for this analysis, *n* = 6 by-pass patients were excluded

In eight cases, a 4-F sheath was used for distal access. In all other cases, a 2.6-F support catheter was the largest access device. In nine cases, a re-entry catheter was used as part of the procedure which failed in two patients.

The average time from the start of the interventional procedure to distal puncture was 75.6 ± 40.6 min with an average total time of 161.9 ± 64.2 min.

### Statistics

Mean values with standard deviation were used to describe continuous variables that passed test for normality (Shapiro-Wilk). Median, range, and proportional values (%) were provided for nominal and ordinal data. Nominal data were compared using the chi-square test. ABI did not pass normality test (Shapiro-Wilk) and was compared using Wilcoxon signed-rank test. The *p* value for statistical significance was set to alpha < 0.05. Analyses were conducted using JMP (JMP 14, SAS Institute Inc.)

## Results

### Technical success

In all cases, a distal vessel was successfully punctured, which indicates a 100% technical success rate for access. The overall technical success rate for CTO crossing was 82% (140/171 of patients). In all cases of successful CTO crossing, the subsequent procedure was finished via an antegrade approach.

Importantly, technical success was significantly reduced in the presence of severe vessel calcification (grade 3), resulting in a success rate of 73.8% (62/82 cases; *p* = 0.01) in severely calcified target lesions. Of note, in a small subgroup of CTO extending over three vascular segments, we observed a trend towards a reduced technical success rate (10 of 14 [71%] patients). Once a through and through wire was established, balloon passage was achieved in all cases, due to the excellent stability of this technique.

Distal access in the distal SFA/P1 segment had a tendency for a higher technical success rate compared with BTK artery access (93.1% vs. 79.6; *p* = 0.06), most likely due to CTO localization in the thigh arteries.

Clinical outcome was assessable in 152 patients with an improvement in 107 (70%) patients. ABI was available in 158 patients before and after the index procedure with a significant improvement of the median ABI from 0.53 (interquartile range 0.39 to 0.61) before the index procedure to 0.85 (interquartile range 0.59 to 1.03) (*p* < 0.001). Pain-free walking distance data were available in 42 cases (majority of cases Rutherford IV to VI), which improved in 39 patients (93%).

### Complications

In total, 13 (7.6%) complications were reported (Table 4). These included groin hematoma, groin bleeding, retroperitoneal hematoma as part of an extended groin hematoma, and renal failure. All of these complications were grade 2 or grade 3 complications based on CIRSE and SIR criteria [21, 22].

**Table 4:** Periprocedural complications

Complications and treatment	Number *N* of 13	Grade*
Groin hematoma	2	2 both cases
Groin bleeding	1	3
1× surgical		
Aneurysma spurium	3	3 all cases
2× compression		
1× surgical		
Retroperitoneal hematoma	2	3 both cases
2× conservative		
Renal failure	1	3
Complications related to distal access		
Hematoma related to distal puncture	2	2 in both cases
Compartment lower thigh	2	2 and 3
1× conservative		
1× surgical		

*Grading according to CIRSE and IRE Guidelines

A total of four complications (4/171 procedures; 2.3%) were related to the distal vascular access which included hematoma at distal puncture site and compartment syndrome of the lower leg. All complications are presented in Table 4. None of the complications occurred when a 4-F sheath was used for distal access. Grade 5 complications were not noted, and there was only one surgical intervention related to distal vascular access with compartment syndrome.

In twelve cases, vascular spasms within the distal access vessel were successfully treated with intraarterial nitroglycerine. In one case, a permanent occlusion of the access vessel (peroneal artery) was observed. However, this was 6 days after the index procedure and combined with an acute in-stent thrombosis of the initial CTO.

Retrograde distal femoral access was not related to any puncture-related complications.

## Discussion

The presented data show that retrograde access is an additional safe option to enhance the success of lesion crossing in complex cases of CTO with only four complications (4/171 procedures; 2.3%) related to the distal puncture. The additional distal puncture and subsequent successful treatment may omit surgery with its associated morbidity and mortality; furthermore, PTA may be the only viable treatment option before amputation [8–11, 23–26]. Our data strengthen the conclusion of the safety aspects for this technique by originating from the largest patient cohort so far supported by good clinical short-term data.

The technical success rate of crossing of 82% was similar to previously published data ranging from 75 to 100%. However, the fact that it is at the lower end of the published range may be related to several factors: Firstly, the retrograde access was used as a bail-out option and mainly used towards the end of an intervention after antegrade approaches had failed (multiple different crossing-wires, endoluminal and subintimal approaches, and even 9 cases of failed re-entry devices). Secondly, the lesions themselves were challenging considering the substantial number of severely calcified lesions within our cohort. Finally, many patients presented with occlusions longer than 20 cm involving the thigh in combination with BTK artery occlusions. These lesions can be classified as TASC D, which are very challenging to treat with an endovascular approach [27].

To our knowledge, the presented work is the first one to assess predictors for technical success using distal retrograde access. We were able to identify the degree of calcification as a predictor of significantly reduced technical success in the most severe calcified lesions (grade 3 by means of PACSS and ACS grading). Previous studies support this finding for antegrade BTK and SFA interventions [28–30] in which calcifications were a predictor of failure in lesion crossing. In one of these studies, analysis of preoperative CT angiography with 100% calcification could be shown to be the best predictor of technical failure in endovascular revascularization of occlusions in the SFA-popliteal region [28]. These findings seem reasonable since calcium is a significant mechanical barrier not only for wire passage but also for device maneuvering. As such, a high calcium load is one of the last truly unsolved challenges for endovascular treatment, not only in terms of lesion crossing but also for long-term patency of the vessel, as the efficacy of most devices and techniques (i.e., plain old balloon angioplasty, stent-assisted angioplasty, drug-coated balloons) is limited by severe calcification [19].

The number of segments occluded showed an effect on technical success which did not reach significance. Of note, despite the large number of patients included in the study, only 14 patients presented with a three-segment occlusion; therefore, the study might be underpowered for any verdict.

The total number of complications (7.6%) was low and mainly related to the access site. In general, this number is similar to previously published data reporting rates of access site complications of up to 13% [14, 31–33]. The additional distal access did not increase the number of serious adverse complications (SAR) as only one case suffered from compartment syndrome requiring surgery. In other words, the access to the groin with 6-F or 7-F sheaths is more prone to complications compared with a small distal access predominantly performed with a support catheter [7]. Distal femoral access seems to be a particularly safe option for SFA-CTO, as we did not observe access-related complications associated with an SFA/P1 puncture in our study. In 12 of 171 cases, transient vascular spasms were observed which were treatable with standard vasodilators. One permanent occlusion of an access site vessel was documented; however, it is questionable whether the occlusion was puncture-related or caused by a concomitant upstream early in-stent thrombosis.

A potential limitation of this study is that distal access was used only after a failed antegrade crossing attempt, which is in contrast to a recent multicenter trial that performed distal access even without a previous antegrade crossing attempt [10]. However, we think it is important to try to cross the lesion from antegrade first and use the distal retrograde approach as bail-out option due to the high success rates using an antegrade approach without the need for an additional distal puncture site. A further limitation is that clinical success and patency data were not available in all patients and limited in most patients to the first 30 days after the index procedure and long-term patency cannot be assessed.

The risk of damage to the distal access vessel should be avoided whenever possible. However, concerning the high technical success and the low complication rate, the additional distal access seems justified not only in CLI patients but also in patients with lifestyle-altering claudication requiring treatment of SFA lesions because it does not appear to pose significant additional risk to those patients. This statement becomes even more convincing because a failed crossing attempt with its subsequent risk for further invasive treatment (e.g., open surgery) may be regarded as a serious complication from the patient’s perspective.

In summary, it can be concluded that distal tibiopedal and distal femoral access sites present a safe and effective means to cross chronic total vascular occlusions at the thigh and below the knee, thereby improving clinical outcome. Technical success decreased with lesion’s degree of calcification and was not correlated with lesion length and Rutherford stage.

## Abbreviations

ABI: Ankle brachial index
ACS: Angiographic calcium score
BTK: Below-the-knee
CIRSE: Cardiovascular and Interventional Radiological Society of Europe
CLI: Critical limb ischemia
CTO: Chronic total occlusions
DSA: Digital subtraction angiography
F: French
HIPAA: Health Insurance Portability and Accountability Act
IRB: Institutional review board
PA: Popliteal artery
PACSS: Peripheral artery calcification scoring system
PAOD: Peripheral arterial occlusive disease
PTA: Percutaneous transluminal angioplasty
SFA: Superficial femoral artery
SIR: Society of Interventional Radiology
